# A Comparative Study of Cutting Electrocautery Versus Scalpel for Surgical Incisions

**DOI:** 10.7759/cureus.107325

**Published:** 2026-04-19

**Authors:** Mahim Koshariya, A. K. Ashish, Sanjay Sisodiya

**Affiliations:** 1 Department of General Surgery, Gandhi Medical College, Bhopal, Bhopal, IND

**Keywords:** cautery, cold scalpel, hemorrhage, incision, ­wound healing

## Abstract

Introduction: The usage of scalpels for surgery started well before the Industrial Revolution, starting from crude instruments to precise and long-lasting instruments. The conventional teaching advocates the usage of scalpels over electrocautery in most teaching hospitals. This was based on a semantic school of thought that existed at that time, which was wary of burn injury to skin, hampered healing, or increased postoperative pain, rather than clinically driven conclusions. Usage of both these instruments is being studied extensively for placement of incisions.

Materials and methods: This study was done in a prospective comparative observational manner among patients on whom an incision was placed with either a scalpel or cutting electrocautery. Patients undergoing surgery in our institution aged 18 to 65 years without any external factors hampering wound healing were included in this study. Patients outside the age group have been excluded. Surgical incisions were placed using a scalpel and cautery and data were recorded. Data were collected using a semi-open questionnaire and interviews and analysis was done using Excel (Microsoft, Redmond, WA, USA).

Results: One hundred twenty-four patients were enrolled, with an average age of 43 years. Cautery was used on 60 patients and a scalpel on 64 patients. Blood loss was significantly higher in the scalpel group (p<0.01). Postoperative pain was measured by visual analogue scoring (VAS) system and it was found that the scalpel group had significantly higher VAS scores on postoperative days three, seven, and 30 (P<0.05). Speed of incision and postoperative wound complication were comparable in both groups. Outcomes of incisions were better with the use of cautery (p<0.05).

Conclusion: Cutting electrocautery may serve as a viable alternative to the traditional scalpel. It is associated with reduced intraoperative blood loss, shorter operative times, and a postoperative course characterized by primary intention healing and a low complication profile.

## Introduction

The field of general surgery, being perpetually dynamic, will troubleshoot its own methods and principles over time, like the appraisal of cautery and scalpels for their merits. The concept of thermal coagulation dates back to 3000 BC when Egyptians used “fire sticks” for the treatment of breast tumours and ulcers [1]. In the Hippocratic era (460-380 BCE), a hot iron rod was used to coagulate bleeding haemorrhoids. Hippocrates also stylised the concept of the surgical knife, which he called “macairion” after small Persian swords [2,3]. During the age of the Roman Empire, Galen and Celsus used small, sharp blades for specialised procedures. They were the first ones to call them “scallpellus”.

The earliest two-piece blade and handle system was designed by Morgan Parker in 1914, which was commercialised with the help of C. R. Bard, a businessman, via the Bard-Parker company. This design was widely received at the American College of Surgeons Clinical Congress in 1915. Bard and Parker used heat sterilisation initially, but it was found to be causing dullness of the blade. They overcame this by using cold sterilisation [4].

The mechanism of action of the scalpel was straightforward. The mechanical action using sharp blades enabled surgeons to place clean cuts employing minimal force, causing less trauma to the surrounding tissues. Perry et al. reported 8% percutaneous injuries caused by a scalpel [5], but in a study from Delhi, only 1.7% patients out of 834 had scalpel-related injuries [6]. A cross-sectional study conducted by Naidu revealed that 16.1% of sharp-related injuries are due to non-bored sharps, such as a scalpel. The common reasons listed include lack of attention, working conditions, and neglect [7].

In the late 1920s, Bovie developed an instrument producing cauterising effects using high-frequency alternating current called the Bovie unit, which was used in over 500 surgeries by Harvey Cushing [8]. The use of cautery necessitates proper application of a grounding pad, evaluation for metallic implants which can form a less resistant grounding pathway for current, deferring the use of alcohol and spirit-based preoperative skin preparations, and exercising caution near supplemental oxygen supply [9]. The thermal injuries thus caused can have medicolegal and ethical implications. The risks related to the emanated smoke are overestimated to produce any significant consequences [10].

Given the historical reliance on the scalpel for skin incisions and the emerging evidence regarding the safety of cutting electrocautery, this study aims to evaluate the clinical outcomes of both techniques. The primary objective is to compare intraoperative blood loss between scalpel and cutting electrocautery incisions. Secondary objectives include the assessment of speed of incision placement, the incidence of postoperative surgical site complications, and the subjective evaluation of postoperative pain using the Visual Analogue Scale (VAS).

## Materials and methods

Study design

This prospective observational study was conducted in the Department of General Surgery, Gandhi Medical College and associated Hamidia Hospital, Bhopal, Madhya Pradesh, to compare the usage and merits of cold scalpel and cutting electrocautery for surgical incisions. The study was conducted from May 2023 to December 2024 on patients visiting the outpatient and emergency departments. For the electrocautery group, a standard electrosurgical generator was set to 'Pure Cut' mode at 30-40 Watts. The active electrode used was a standard needle-tip blade.

Inclusion and exclusion criteria

Patients who underwent surgery with a surgical incision for emergency or elective indications and who were aged 18 to 65 years were included in this study. Patients were excluded if they had prior surgery at the same site, were undergoing anticoagulant or corticosteroid therapy, or had anemia, uncontrolled diabetes mellitus, active wound infections, or other health conditions that might impact wound healing. Additionally, those who received local anesthetics intraoperatively or perioperatively, as well as individuals younger than 18 or older than 65 years, were not included.

Data collection

After obtaining approval from the ethical committee (Institutional Ethical Committee, Gandhi Medical College, Bhopal; Approval No. 99/IEC/2023) data was collected by interviewing the patients with a semi-open questionnaire and informed consent. The semi-open questionnaire was validated for internal consistency and focused on subjective pain reporting, surgical scar satisfaction, and mobility scores. The necessary investigations for the study have been obtained from the patient's record.

Statistical analysis

The data collected was entered into Microsoft Excel (Redmond, WA, USA) and jamovi for statistical analysis. The primary outcome of the study was intraoperative blood loss, while the secondary outcomes included speed of incision placement, postoperative pain, and outcome of incision. Continuous and ordinal variables were expressed as mean ± standard deviation, and categorical variables as frequencies and percentages. Patient records with incomplete subjective data for specific long-term postoperative follow-up intervals (e.g., missing VAS pain evaluations or unrecorded wound checks at day 30) were treated as lost to follow-up for those specific time points. These missing data points were omitted via listwise deletion for interval-specific comparative calculations, though the patients' available early postoperative data were retained for overall analysis.

To compare the continuous variables, inferential statistical tests were applied. Given the presence of skewed distributions and ordinal data (VAS), the non-parametric Mann-Whitney U test was utilized. To determine not only the statistical significance but also the clinical magnitude of these differences, the effect size (r) was calculated for all comparisons using the formula r = Z/ √ N where Z is the standardized test statistic and N is the total sample size. Effect sizes were interpreted using standard thresholds: r < 0.3 (Small), 0.3</= r < 0.5 (Medium), and >/=0.5 (Large). To further evaluate the relationship between wound size and the efficacy of the surgical modality, a multiple linear regression model was constructed. Intraoperative blood loss was set as the continuous dependent variable, while incision length, method(electrocautery versus scalpel), and an interaction term between the two were included as independent predictors to determine if the rate of blood loss per centimeter of incision varied significantly between the two methods. 

To systematically evaluate the impact of incision size on the efficacy of the chosen surgical instrument, a stratified subgroup analysis for blood loss was conducted. The data was categorized using two distinct splitting methods.

Tertile Stratification

Incision lengths were divided into three groups based on the 33rd and 66th percentiles to observe progressive trends (Short: </=5 cm, Medium: > 5 to </= 13.8 cm, Long: > 13.8 cm).

Median Split Stratification

A binary categorization was performed based on the median incision length to compare smaller versus larger wounds directly (Shorter: </=6.0 cm, Longer: > 6.0 cm).

Within each predefined stratum, the Mann-Whitney U test and subsequent effect size (r) calculations were performed to determine if the magnitude of superiority of one modality over the other was dependent on the extent of the surgical incision. For all statistical tests, a two-tailed p-value of < 0.05 was considered statistically significant.

## Results

Of the 124 participants, there were 51 males and 73 females. The mean age of all participants was 43 years. The age of subjects had a standard deviation (SD) of about 13.8 years among females and 14.7 years among males, the mean age of each of them being 44.5 years and 40.3 years, respectively.

**Figure 1:**
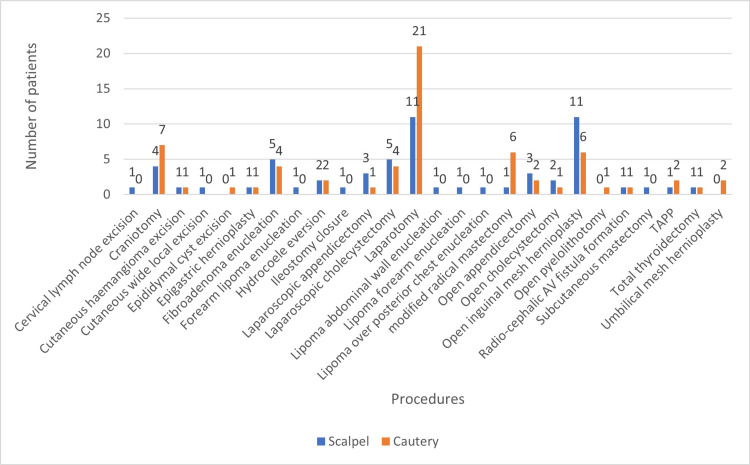
Number of incisions by scalpel and cutting electrocautery TAPP - laparoscopic transabdominal preperitoneal hernioplasty

The mean blood haemoglobin value was 11.3g/100mL, which was more or less the same as that among both sexes and both groups. A similar trend can be observed in white cell counts, which averaged at 8000/mm^3^, and platelet counts, averaging at 3.8 *103/mm^3^. A total of 124 procedures have been observed in this study. Most procedures scrutinised were laparotomy (32), open inguinal mesh hernioplasty (17), followed by craniotomy (11). Laparotomy was a singular procedure that has been conducted in the largest number by scalpel, but in the cautery group, the position is shared by open inguinal mesh hernioplasty and laparotomy. A scalpel was used on 64 patients, and cutting electrocautery was used in 60 patients (Figure 1).

The length of incisions placed was measured, with a mean length of 10.1 cm. Cautery was used to place incisions averaging around 8.4 cm, and a scalpel 11.7 cm. Time taken for incision placement was measured using a stopwatch; the average time taken was 7.03 s. While cautery took 5.8 s on average, the scalpel utilised 8.2 s. While comparing the speed of the incision placement, the average speed with which incisions were placed was around 1.3 cm/s for both groups.

**Figure 2:**
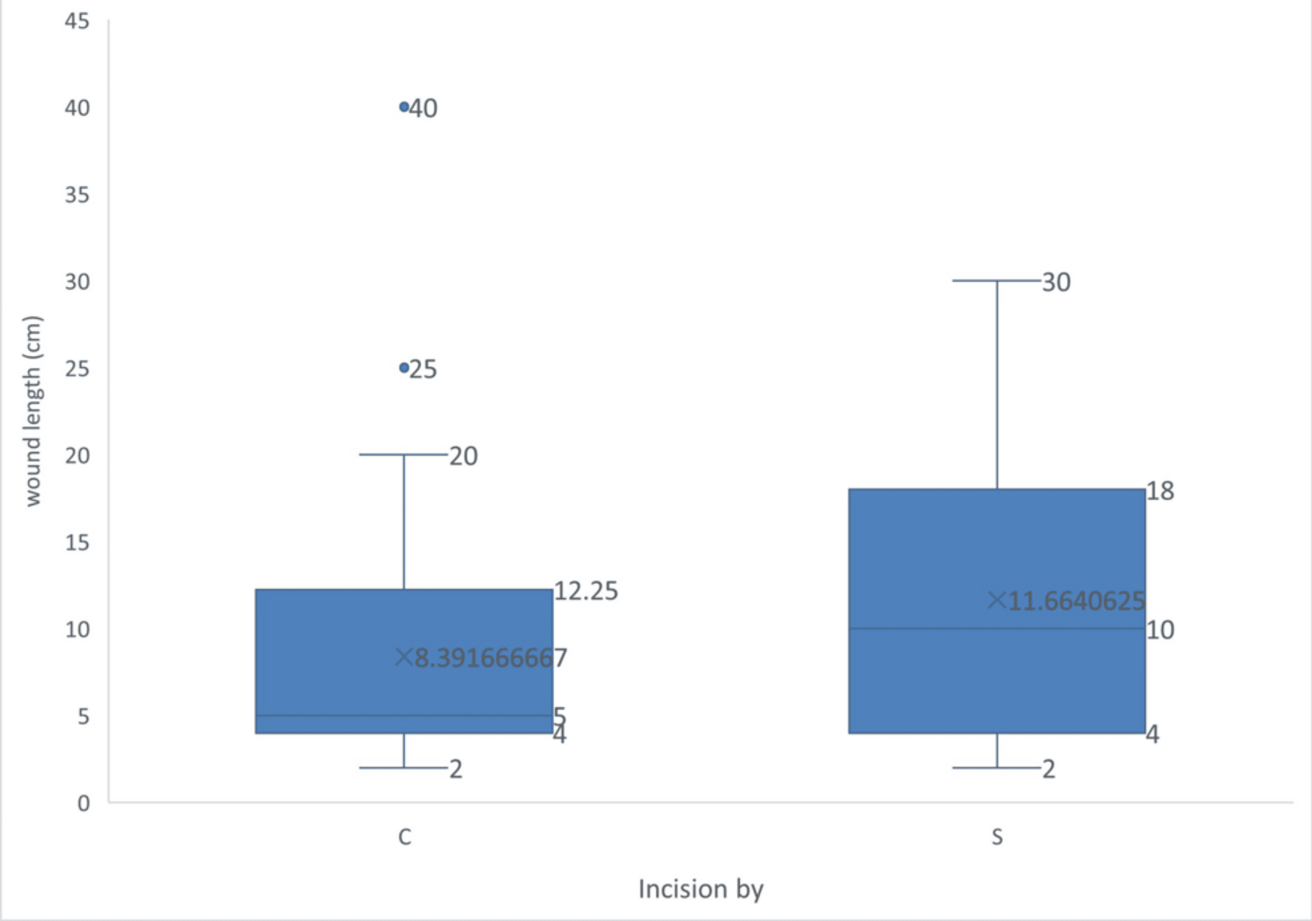
Wound length distribution C - Cutting electrocautery S - Scalpel

The mean incision length was significantly shorter in the electrocautery group compared to the scalpel group (8.39 ± 7.22 cm vs 11.66 ± 8.29 cm; p = 0.021). Given the potential for incision length to act as a confounding factor in blood loss, further stratified analysis was performed using both tertiles (≤5 cm, 5-13.8 cm, >13.8 cm) and median split (≤6 cm and >6 cm). The tertile distribution included 55 patients in the short, 27 in the medium, and 42 in the long incision groups, while 55 and 69 patients fell below and above the median, respectively (Figure 2).

**Figure 3:**
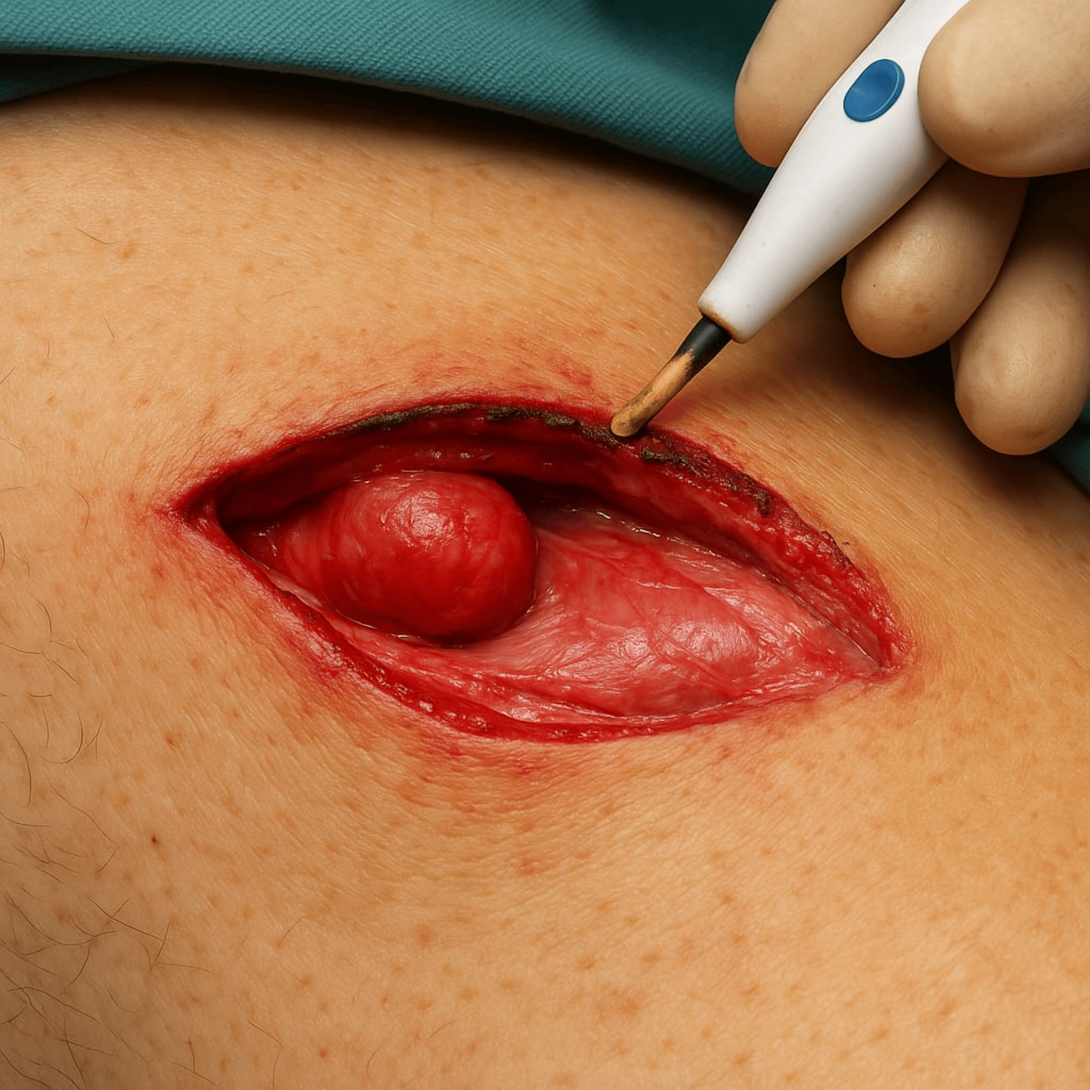
Right hernioplasty incision by cutting electrocautery

**Figure 4:**
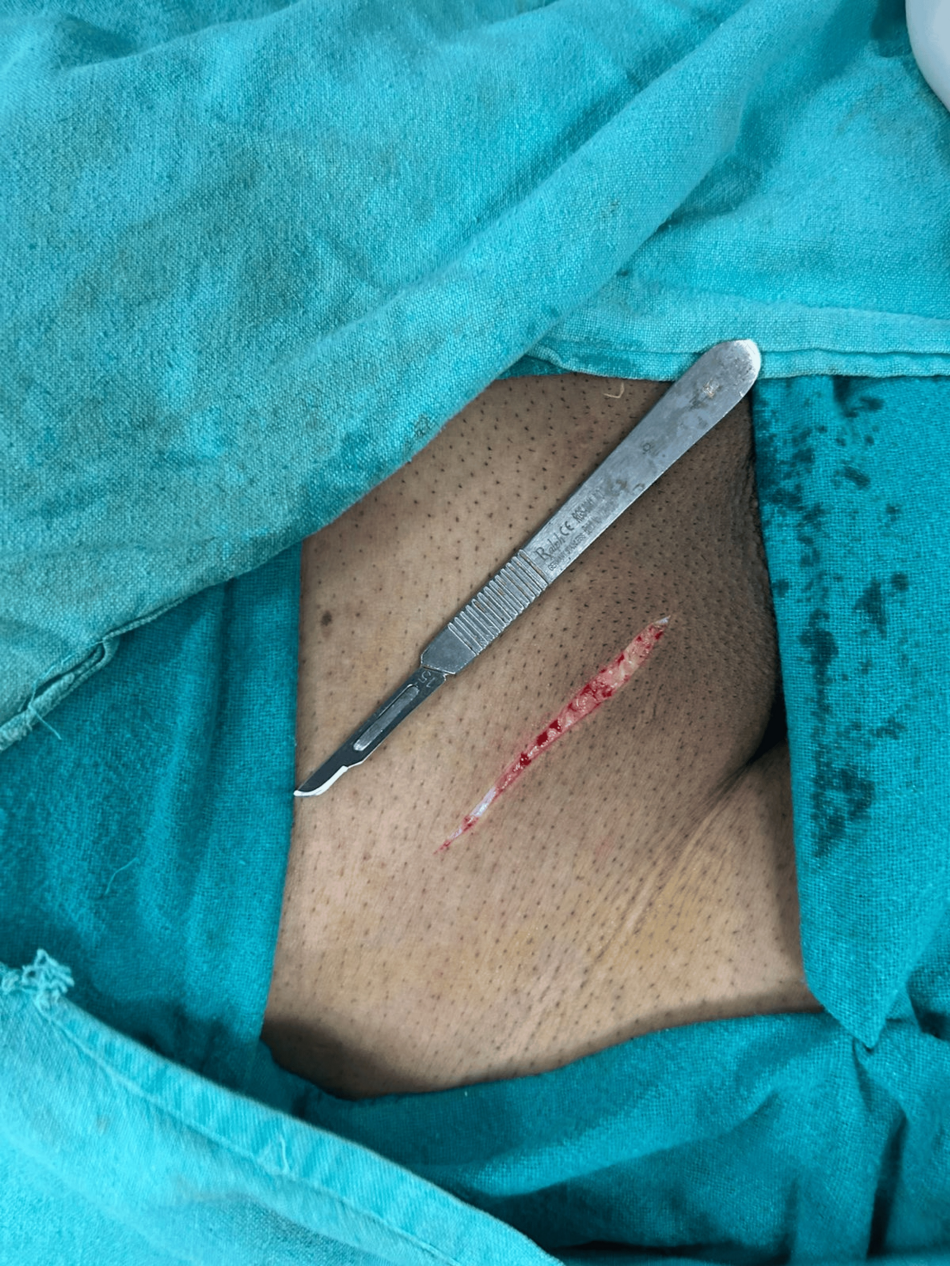
Right hernioplasty incision by scalpel

**Figure 5:**
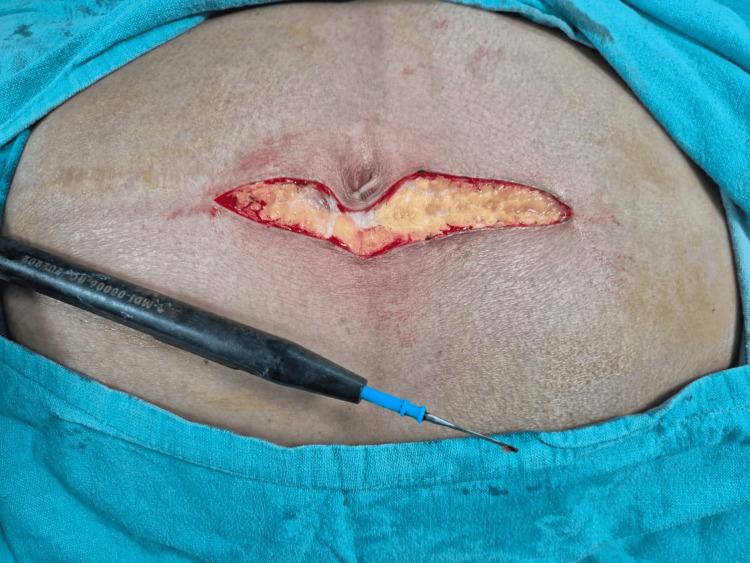
Laparotomy incision by cutting electrocautery

**Figure 6:**
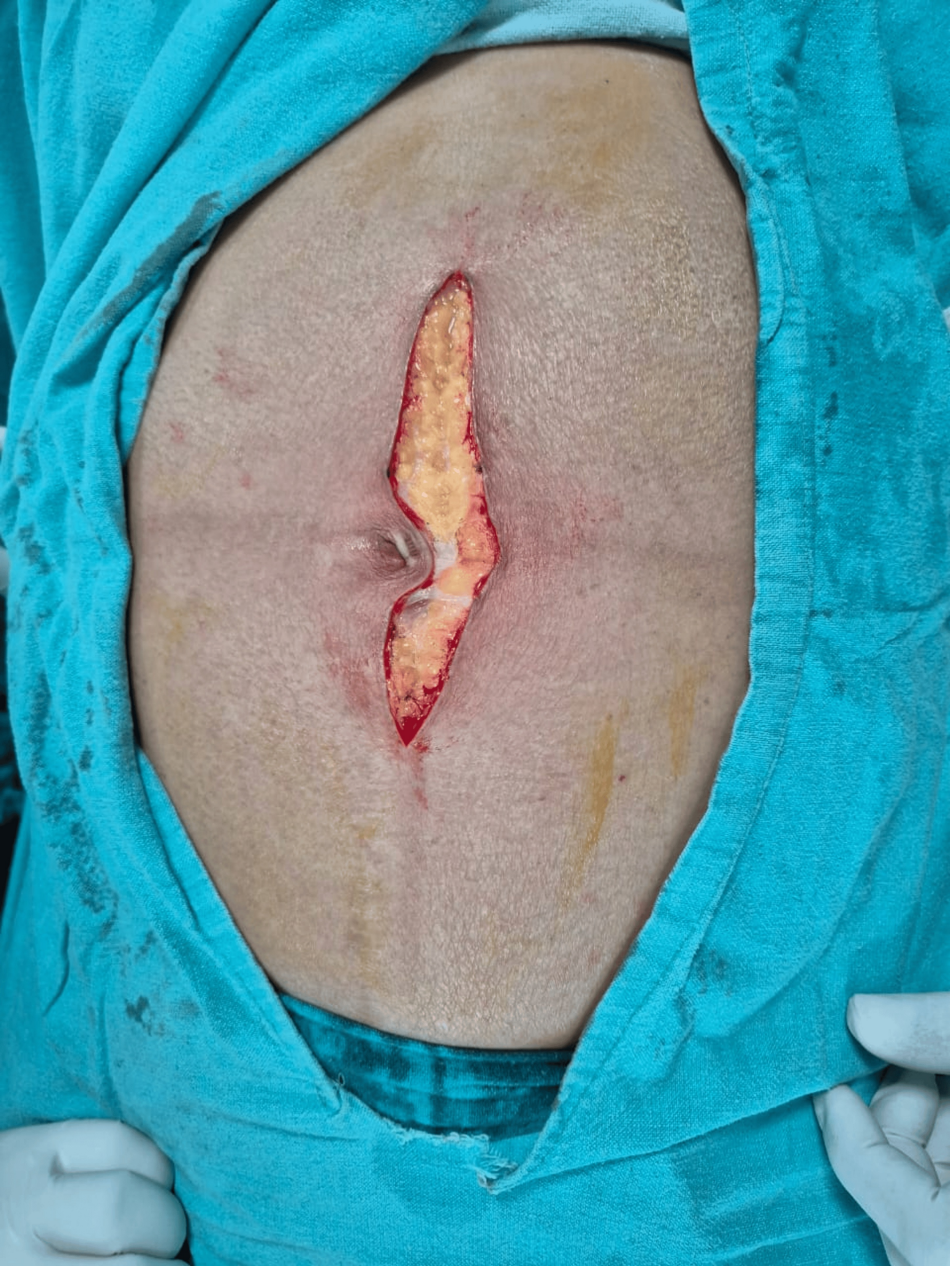
Laparotomy incision by scalpel

Among the patients on whom the scalpel was used mean blood loss was 2.29g, while only 1.07g blood loss was recorded in the cautery group. Standard deviation for both groups was 0.7g and 2.07g, respectively. Mean blood loss was significantly higher in the scalpel group compared with the electrocautery group (2.29 ± 0.70 g vs 1.07 ± 2.07 g: p=<0.001, r=0.392). Incisions made by cautery and scalpel are given below (Figures 3-6).

Across tertile-based stratification (Table 1), mean incision lengths between electrocautery and scalpel groups were comparable within each stratum (p = 0.92, 0.464, and 0.965 for long, medium, and short groups, respectively), indicating adequate matching of incision length. Despite this, blood loss remained significantly lower in the electrocautery group in both long (1.70 ± 0.74 vs 3.52 ± 2.44; p = 0.001) and medium (1.22 ± 0.66 vs 2.08 ± 0.92; p = 0.012) incision groups, while the difference in the short incision group did not reach statistical significance (p = 0.18). Similarly, on median-based stratification (Table 1), incision lengths between groups were not significantly different (p = 0.511 and 0.743 for longer and shorter groups, respectively). However, blood loss was significantly lower in the electrocautery group among patients with longer incisions (1.45 ± 0.74 vs 3.06 ± 2.19; p = 0.00027), whereas no significant difference was observed in the shorter incision group (p = 0.064).

**Table 1:** Tertile stratification and median split analysis of blood loss across incisions of different size C - Cutting electrocautery S - Scalpel

	LONG	MEDIUM	SHORT	LONGER	SHORTER
C	1.7+/-0.74	1.22+/-0.66	0.74+/-0.41	1.45+/-0.74	0.74+/-0.41
S	3.52+/-2.44	2.08+/-0.92	0.92+/-0.46	3.06+/-2.19	0.92+/-0.46
MEAN	2.91+/-2.21	1.63+/-0.91	0.81+/-0.44	2.41+/-1.92	0.81+/-0.44
P VALUE	0.001	0.012	0.183	0.00027	0.064

These findings suggest that the reduction in blood loss associated with electrocautery persists across comparable incision lengths, particularly in medium and long incision groups, indicating that the observed effect is not solely attributable to differences in incision length.

**Figure 7:**
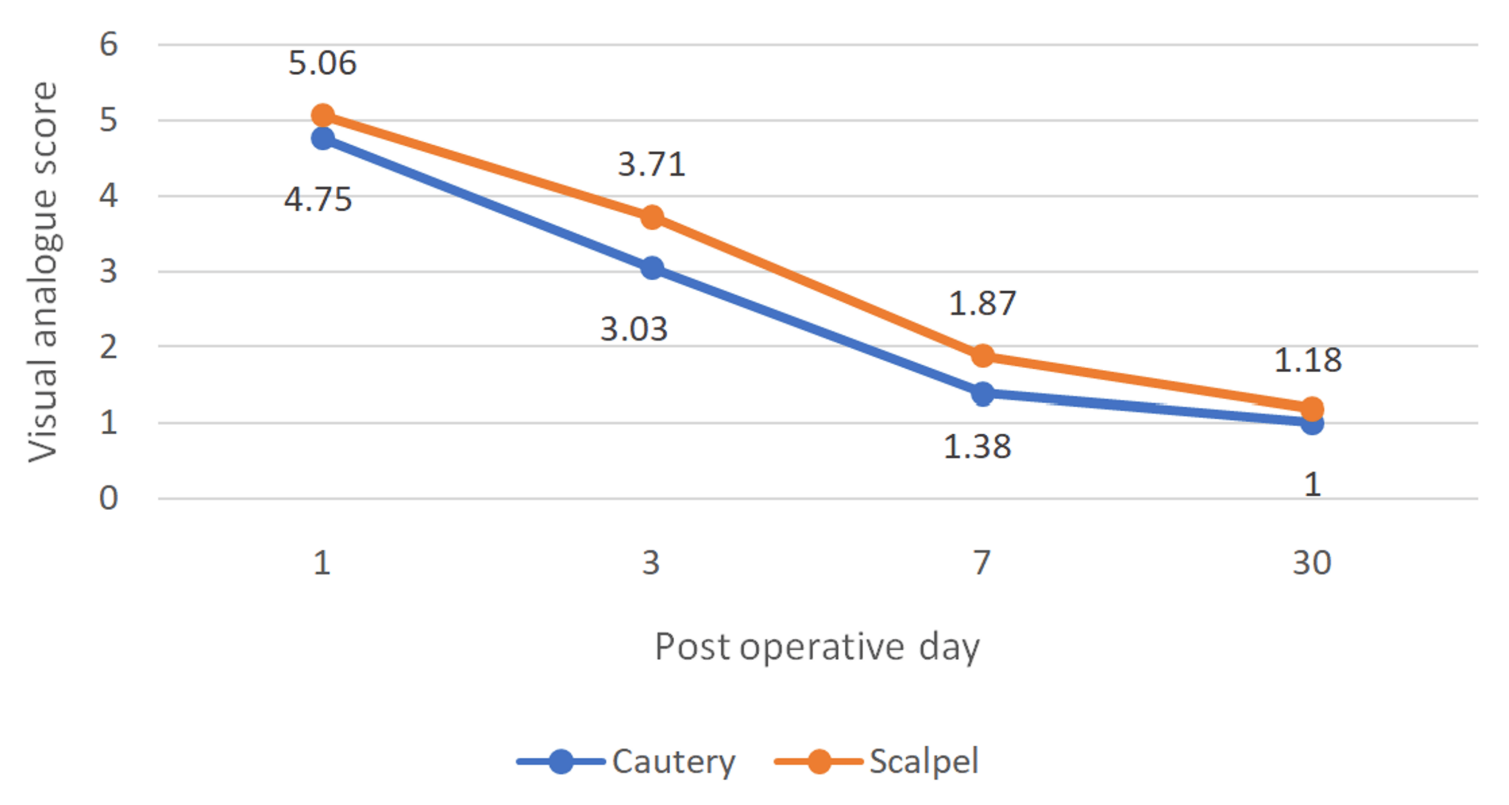
Comparison of mean postoperative VAS score for both groups VAS - Visual Analogue Scale

Postoperative pain was evaluated on day one, three, seven, and 30 using VAS. The mean VAS scores were compared for both groups (Figure 7). 

There is an apparent trend of decreasing VAS with time evident in both groups. On postoperative day one, pain scores were comparable between the two modalities. The mean VAS score for the electrocautery group was 4.75 ± 1.34, while the scalpel group reported a mean score of 5.07 ± 0.90. This difference was neither statistically significant (p = 0.331) nor clinically meaningful, demonstrating a negligible effect size (r = 0.08). On postoperative days three and seven, patients whose incisions were made using cutting electrocautery reported statistically significantly lower pain scores compared to those in the scalpel group. On day three, the electrocautery cohort demonstrated a mean VAS score of 3.03 ± 1.55 versus 3.71 ± 1.69 for the scalpel cohort (p = 0.025). This trend was maintained on day seven, with the electrocautery group recording a mean score of 1.38 ± 1.11 compared to 1.87 ± 1.24 for the scalpel group (p = 0.002). While these reductions in pain for the cautery group are statistically significant, the calculated effect sizes for day three (r = 0.20) and day seven (r = 0.22) indicate that the clinical magnitude of the difference is relatively small. The mean VAS values were consistently higher in the scalpel group, although a significant association could only be established on postoperative days three, seven, and 30. By post-operative day 30, surgical site pain had nearly entirely resolved in both cohorts. The electrocautery group reported a uniform baseline score indicating a complete absence of pain (1.00 ± 0.00), whereas the scalpel cohort exhibited minimal residual pain (1.19 ± 0.49). Although this minor discrepancy achieved statistical significance (p = 0.014), the practical clinical effect remains minimal (r = 0.12).

The incision was observed in the postoperative period for any surgical site infections (SSI), on postoperative days three, seven, 15, and 30 (Table 2). On postoperative day seven, pus formation was observed in 8.1% patients in the scalpel group compared to 1.7% patients in the electrocautery group. Although the incidence was higher in the scalpel group, this difference was not statistically significant (Fisher's exact test, p=0.21). The relative risk of pus formation with scalpel incision was 4.84 (95% CI 0.58-40.21). On postoperative day 15, wound complications (pus formation or burst abdomen) were observed in 11.3% of patients in the scalpel group compared with 1.7% of patients in the electrocautery group. The difference was not statistically significant (Fisher's exact test, p=0.06). The relative risk of wound complications with scalpel incision was 6.66 (95% CI 0.85-52.1).

**Table 2:** Postoperative assessment of wound for SSI SSI - surgical site infection

	Day 3	Day 7		Day 15			Day 30
Incision by	Normal	Normal	Pus	Normal	Pus	Burst abdomen	Normal
Cautery	60	59(98.3%)	1(1.7%)	58(98.3%)	1(1.7%)	0	36
Scalpel	64	57(91.9%)	5(8.1%)	55(88.7%)	6(9.7%)	1(1.6%)	44
Total	124	116	6	113	7	1	81

The outcome of incision was determined on the day when further follow-up for wound management was not required, which never crossed 30 days in most patients. Of the total 58 patients assessed in the cautery group, one (1.7%) patient required secondary suturing for wound dehiscence. Of the 61 patients in the scalpel group, eight (13.1%) patients required secondary suturing for wound dehiscence. This difference was statistically significant (Fisher's exact test, p=0.0196) with a relative risk of 7.7 (95% ci 1.0-58.2). The remaining wound healed by primary intention.

## Discussion

**Figure 8:**
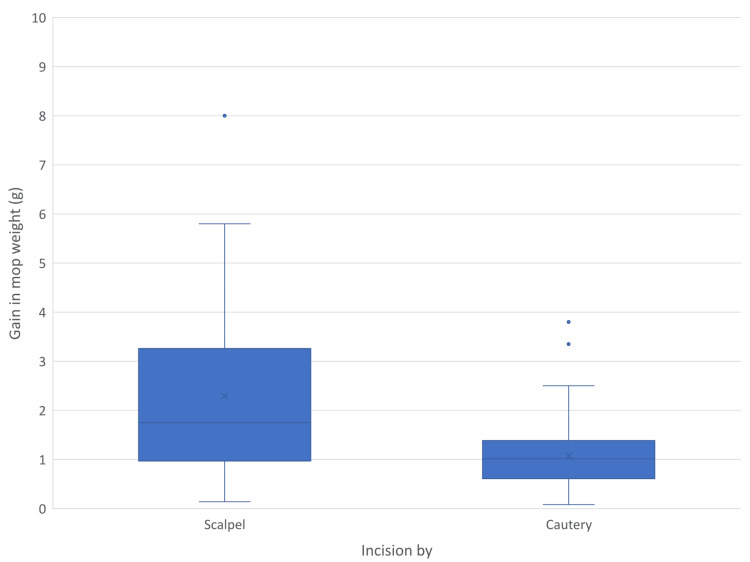
Blood loss calculated by gain in mop weight

Figure 8 shows a comparison of blood loss estimated by the gain in mop weight in both groups. The mean of the cautery group is significantly lower than that of the scalpel group (95% CI 0.66-1.78).

Prakash et al. conducted a randomised controlled trial where they compared mean blood loss using scalpel and cautery, where the mean blood loss was found to be 6.46mL ± 3.94mL and 23.40mL ± 15.28mL, respectively [11]. This difference was found to be statistically significant (p=0.0001). Kearns et al. in 2001 did a prospective randomised trial where they found that blood loss per square area of the wound is less in the cautery group, 0.8 +/- 0.1, than the scalpel group, 1.7+/-0.3 ml/cm^2^; P = 0.002 [12]. Ismail et al. conducted a systematic literature review and meta-analysis on the comparison between surgical incisions made with a scalpel and cutting electrocautery in 2017. Forty-one studies, including 36 randomized controlled trials (RCTs), were reviewed [13]. They concluded that incisions with cutting electrocautery are having lesser standard mean deviation (SMD), -1.16, 95% CI [-1.60 to -0.72]. Talpur et al. compared incisions made by scalpel and cautery in 283 patients; they found that the cautery group had lesser blood loss per unit area than the scalpel, 1.8262 mL/cm^2^ and 1.1346 mL/cm^2^ [14].

**Figure 9:**
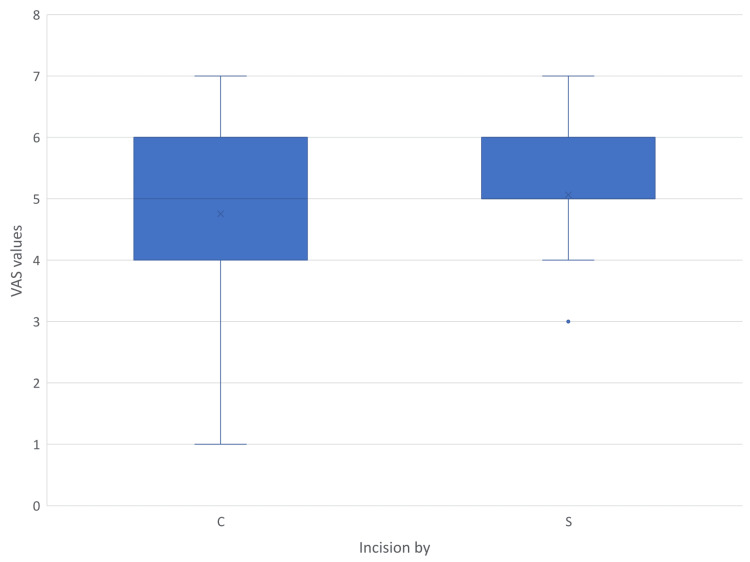
VAS score recorded on postoperative day one C - Cutting electrocautery S - Scalpel VAS - Visual analogue Scale

**Figure 10:**
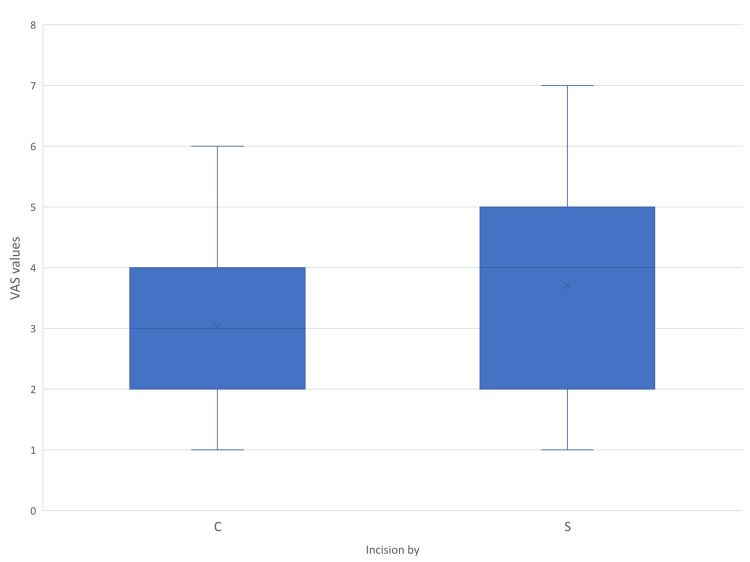
VAS scores recorded on postoperative day three C - Cutting electrocautery S - Scalpel VAS - Visual analogue Scale

**Figure 11:**
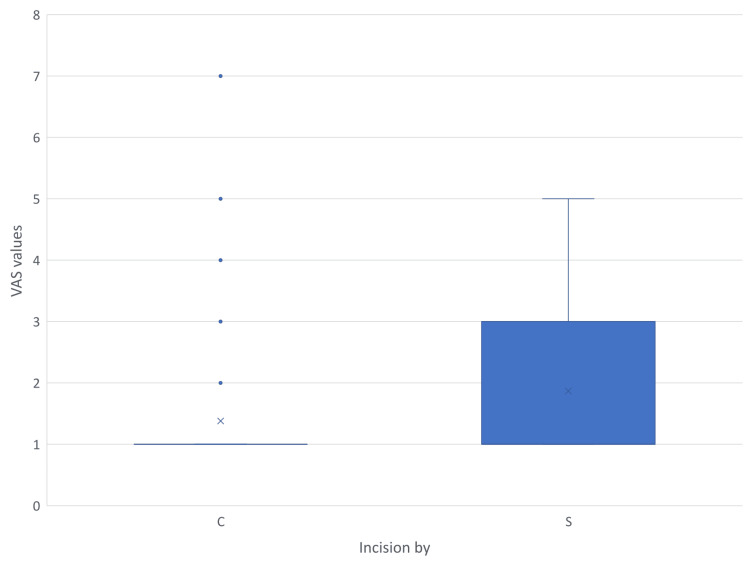
VAS scores recorded on postoperative day seven C - Cutting electrocautery S - Scalpel VAS - Visual analogue Scale

**Figure 12:**
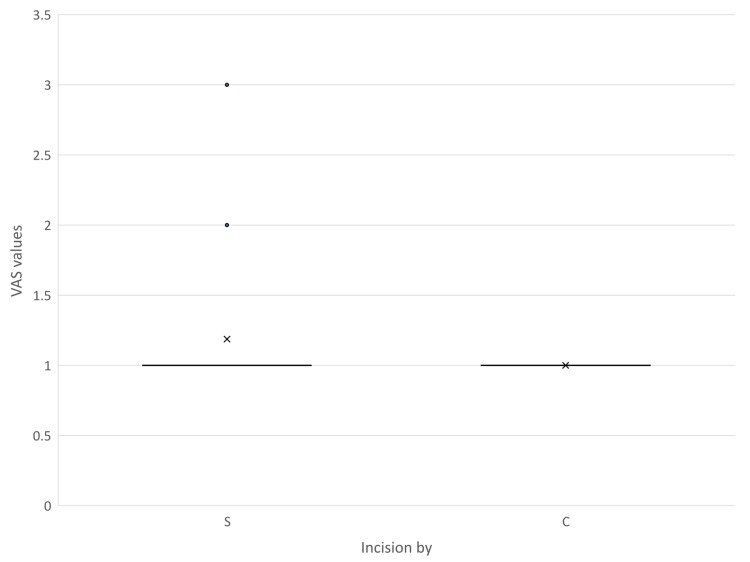
VAS scores recorded on postoperative day 30 C - Cutting electrocautery S - Scalpel VAS - Visual analogue Scale

Pain borne due to the wound in the postoperative period was assessed using VAS. The findings are shown in Figures 9-12. Mann-Whitney U test showed a time-dependent pattern, with no significant difference on day one, but modest, statistically significant reductions favouring electrocautery on days three, seven, and 30. Analysis showed that Incisions placed by scalpel and cautery have almost the same chance of having a VAS value more than 97.5% population on postoperative day three (95% CI = -0.10-0.72). On day three, cautery is 0.6 times less likely to cause pain in more than 97.5% population than using a scalpel (95% CI = 0.08-1.28), and using cautery is 0.39 times less likely to cause pain in more than 97.5% population on day seven (95% CI = 0.08-0.90). On postoperative day 30, all subjects in the cautery groups had a VAS score of one, the same being for the majority of the scalpel group.

Multiple outliers can be seen on day seven in the cautery group, indicating extreme values above the upper limit. In all illustrations mean VAS values of the scalpel group were higher than those of the cautery group. Since the VAS scores recorded in the postoperative period have data lacunae for some patients due to reasons like sedation and lost to follow-up, regression analysis becomes complex and biased due to reduced sample size in individual subsets. 

**Figure 13:**
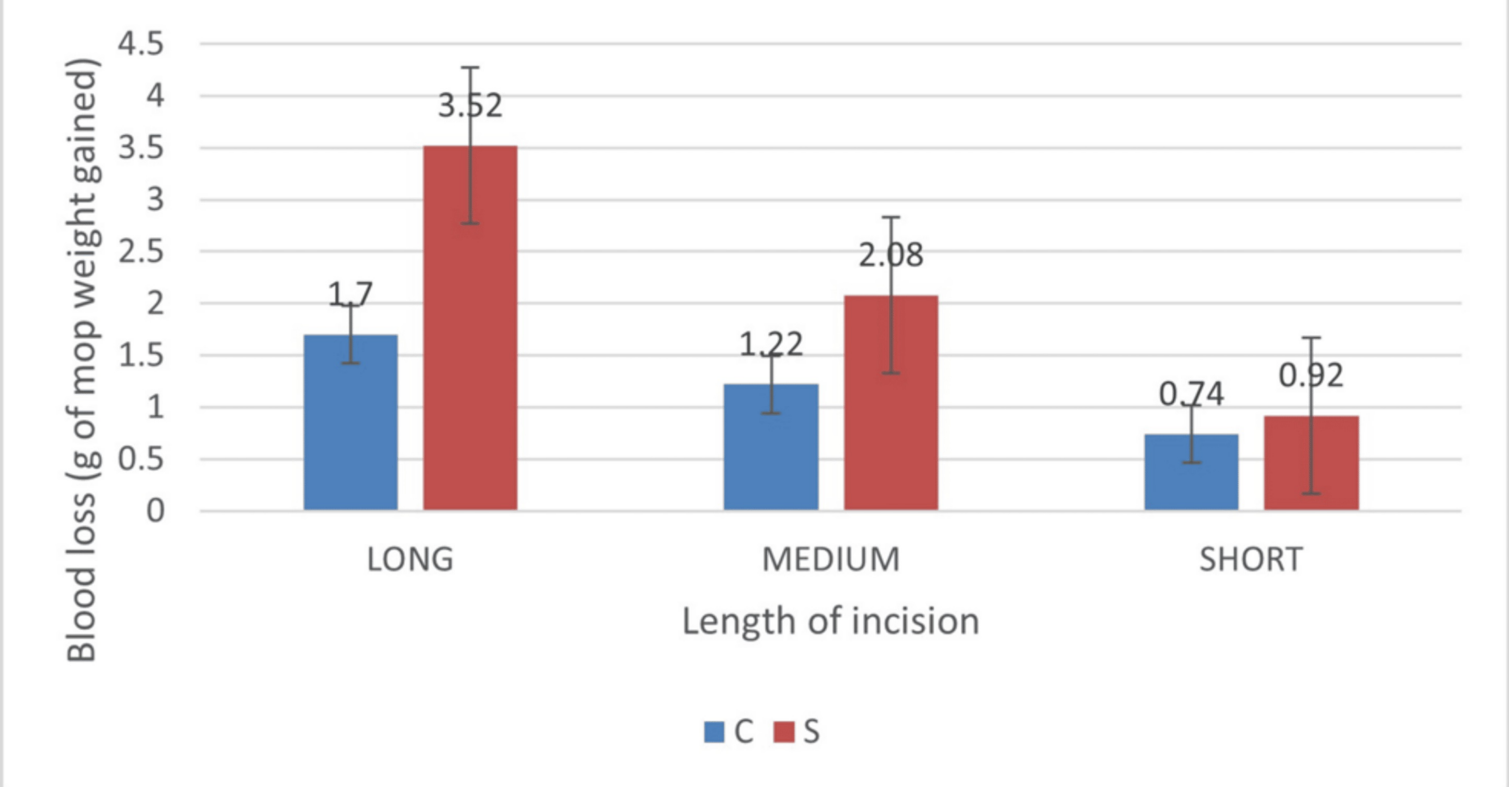
Blood loss across tertiles C - Cautery S - Scalpel

The present study demonstrated a significant reduction in intraoperative blood loss with the use of electrocautery compared to scalpel. Although the mean incision length was significantly shorter in the electrocautery group, raising the possibility of confounding, this was systematically addressed through both stratified analyses and multivariable regression. Notably, stratification by incision length using tertiles and median split revealed that incision lengths were comparable between groups within each stratum, while the reduction in blood loss with electrocautery persisted, particularly in medium and long incision groups. This consistency across strata suggests that the observed benefit of electrocautery is not merely a function of shorter incision length but reflects an independent effect of the incision modality itself (Figure 13).

Furthermore, the effect was more pronounced in longer incisions, where the potential for blood loss is inherently greater, thereby reinforcing the clinical relevance of electrocautery in procedures involving larger operative fields. The absence of a statistically significant difference in shorter incisions may be attributable to lower baseline blood loss and reduced power to detect small differences in this subgroup.

The mean incision length placed by either group is 8.3 cm in the cautery group and 11.7 cm in the scalpel group. This difference was statistically significant, p=0.02. Both the incision method and incision length were significant independent predictors of blood loss. The multiple linear regression model ran was statistically significant, F(2, 121) = 37.56, p < 0.001, explaining approximately 38.3% of the variance in surgical blood loss (R^2^= 0.383; Adjusted R^2^= 0.373). For every 1 cm increase in the length of the incision, there was a corresponding average increase in blood loss of 0.107 g (p < 0.001), regardless of the instrument used. The interaction between the instrument and the wound length was highly significant (p = 0.003). This proves mathematically that the rate of blood loss per cm of incision is significantly slower when using electrocautery compared to a scalpel. Specifically, for every 1 cm increase in the wound, the electrocautery group lost 0.09 g less than the scalpel group. These findings indicate that both the method of incision and the incision length contribute meaningfully and independently to the blood loss.

There were variations in mean wound length and intraoperative blood loss-quantified strictly in grams of mop weight gained-alongside differing outcomes when comparing electrocautery and scalpel incisions. In the most frequently performed procedure, right inguinal hernia mesh hernioplasty (n=11), the mean wound length was 4.64 cm with an overall mean blood loss of 1.19 grams; electrocautery yielded a lower mean blood loss (1.00 grams, n=5) compared to the scalpel (1.35 grams, n=6), though not statistically significant (p=0.329).

For right breast fibroadenoma enucleation (n=6, mean length: 3.08 cm, overall loss: 0.61 grams), electrocautery incisions resulted in less than half the blood loss of scalpel incisions (0.35 vs. 0.87 grams; p=0.376). Open appendicectomies (n=5, mean length: 5.60 cm, overall loss: 1.23 grams) followed a similar trend, where the electrocautery cohort averaged 0.97 grams of blood loss compared to 1.63 grams with the scalpel (p=0.200). Notably, in major neurosurgical interventions involving craniotomies (e.g., for hematoma evacuation or tumor excision; n=11), the average wound length was 15.45 cm with the highest overall blood loss in the cohort at 3.55 grams. Within these high-blood-loss craniotomy cases, electrocautery (n=4) substantially reduced mean blood loss to 2.39 grams compared to 4.21 grams when the scalpel (n=7) was utilized (p=0.230). Conversely, in minimally invasive procedures such as laparoscopic cholecystectomy for calculous cholecystitis (n=5, mean length: 2.80 cm, overall loss: 1.00 grams), blood loss was marginal between electrocautery (1.05 grams) and scalpel (0.93 grams; p=0.800). Laparoscopic appendicectomies (n=4, mean length: 2.00 cm, overall loss: 0.88 grams) and left inguinal hernia repairs (n=4, mean length: 4.75 cm, overall loss: 0.74 grams) exhibited minimal baseline blood loss, precluding robust comparison due to limited scalpel usage in these subsets

**Figure 14:**
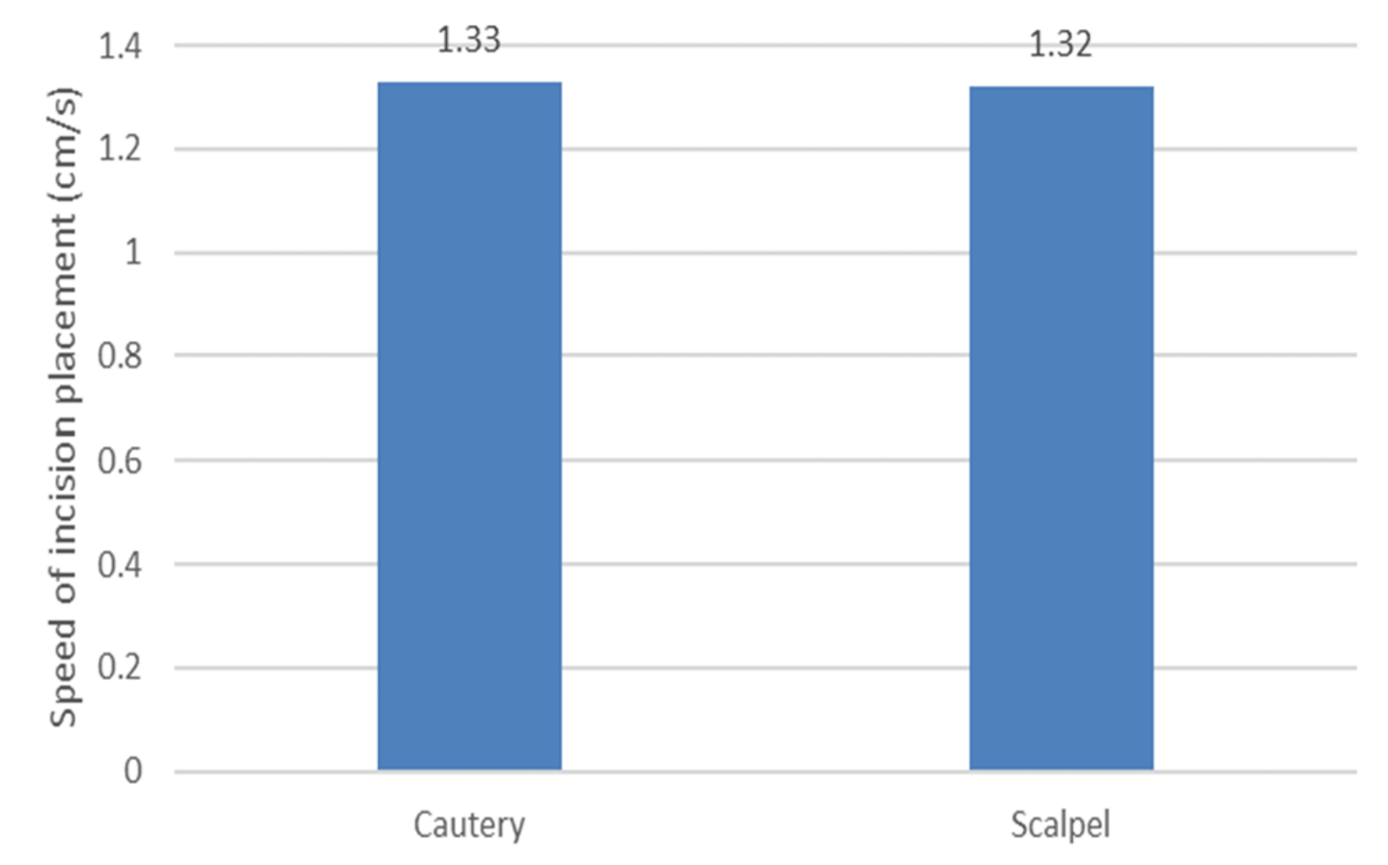
Speed of incision placement in both groups cm/s - centimeter per second

The time taken to place a unit length of incision was determined and compared (Figure 14). The absence of a stark difference is endorsed by the dearth of any significant difference among them.

In multiple RCTs comparing electrocautery and scalpel, it was shown that cautery can place incisions faster [12,14-16]. In a heterogeneous cohort studied by Ayandipo et al. similar findings were obtained. But in an RCT comparing scalpel and electrocautery, the speed with which incision was placed was comparable [17]. 

**Figure 15:**
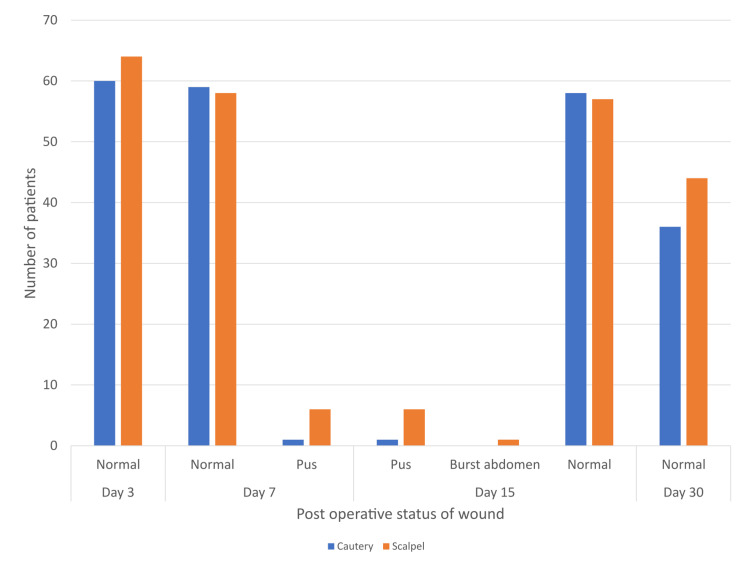
Features of surgical site infection during the postoperative period Day - postoperative day Cautery - cutting electrocautery Pus - pus formation

Of all 124 patients, 15 patients had complicated wounds (Figure 15). 

There was a higher occurrence of wound complications in the scalpel group, although there was no statistically significant difference among them. The scalpel group is more likely to have wound complications, RR=6.66.

Prakash et al. in 2015 conducted an RCT on 82 patients comparing the midline laparotomy incision with scalpel and electrocautery. They found that electrocautery incisions had comparable postoperative complications [11]. Franchi et al. in 2001 conducted a study where they compared the midline laparotomy incisions placed with diathermy and scalpel on 964 patients with uterine malignancy, and an appraisal for early and late postoperative complications found them to be comparable [18]. An RCT was conducted on 133 women with gynaecological tumours by Rongetti et al. in 2014, comparing incisions made by scalpel and electrocautery for the occurrence of surgical site infections. They found that there is no significant difference in the occurrence of SSI among them [19]. Eren et al. in 2013 conducted an RCT to compare midline abdominal incisions using a scalpel and electrocautery for postoperative complications and found that both have comparable early and late postoperative complications [20]. The assessment of SSI was conducted by a resident surgeon who was blinded to the incision method used.

**Figure 16:**
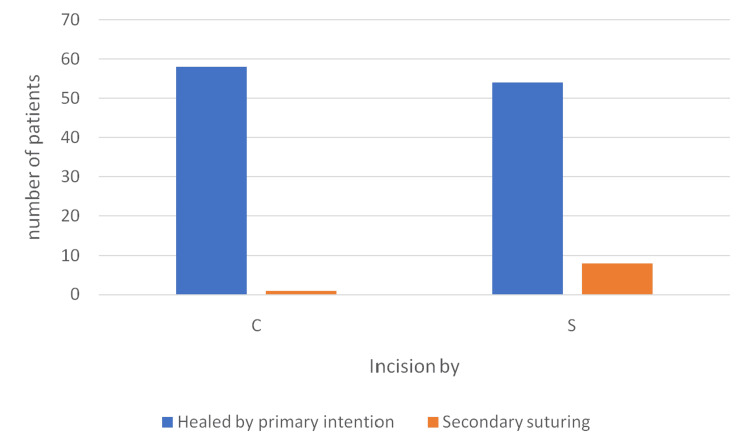
Outcome of incision in both groups C - cutting electrocautery S -scalpel

In total, 10 patients either required secondary procedures or died due to complications (Figure 16).

The obvious distinction between the two groups is supported by Fisher's exact test, which showed a significant difference between them (p-value <0.05). The remaining 112 patients’ wounds healed by primary intention, and follow-up discontinued for wound as per the surgeon’s discretion. 

While the vast majority of surgical incisions (90.3%) healed by primary intention without adverse events, 10 cases (8.06% of the overall cohort) developed localized or severe wound complications within the 30-day postoperative period. A marked disparity in complication rates was observed between the two incision modalities: electrocautery was associated with a markedly lower complication rate of 1.7% (one of 60 procedures) compared to the traditional scalpel, which yielded a complication rate of 14.1% (nine of 64 procedures). The incidence of SSIs - clinically evident as purulent discharge or erythema predominantly occurring on day seven or day 15 - was strongly clustered among high-risk, contaminated, or extensive surgical interventions. Specifically, exploratory laparotomies for intestinal obstruction (including adhesiolysis, bowel resection, and ileostomy formations) accounted for 60% (n=6) of all complicated cases. In these instances, where a scalpel was exclusively utilized, the surgical sites failed to heal by primary intention and uniformly necessitated secondary suturing. Major resections, including open splenectomies (15 cm wound length) and radical cholecystectomies (25 cm wound length), also suffered purulent complications requiring secondary closure. The solitary complication observed in the electrocautery group occurred following an extensive wide local excision for squamous cell carcinoma of the scrotum (25.0 cm wound length), which developed purulent discharge leading to wound dehiscence and required secondary suturing.

Due to the highly diverse nature of the surgical interventions, individual procedure types lacked the requisite sample size to power isolated tests of significance for SSIs. To facilitate robust statistical analysis, procedures were stratified by clinical severity into major exploratory laparotomies (n=28) versus all other procedural interventions (n=96). Among the major exploratory laparotomies - which inherently carry a higher baseline risk for contamination and wound morbidity - the use of a scalpel was associated with a clinically substantial complication rate of 36.8% (seven of 19 cases), whereas electrocautery yielded a 0% complication rate (0 of nine cases). A Fisher’s exact test demonstrated a strong trend toward statistical significance for this disparity (p = 0.0621). Conversely, in the subgroup comprising all other routine surgical interventions, overall complication rates remained low and did not differ significantly between the electrocautery (1.9%; one of 51) and scalpel (4.4%; two of 45) modalities (Fisher’s exact test, p = 0.598). These data suggest that the protective hemostatic and thermal benefits of electrocautery regarding wound healing are most clinically pronounced in extensive, high-risk abdominal procedures.

This study has limitations that, when rectified, might produce a result with much more conviction. The blood loss was assessed by the difference in mop weight rather than in a direct and objective manner. Pain assessment was done using VAS scoring, which is highly subjective. The incidence of wound complications is small, limiting statistical power. Additionally, the relatively smaller sample size within subgroups, particularly in the medium incision group, may limit the statistical power to detect differences across all subgroups. Matching between the groups in terms of wound length was not done, although linear regression attributes influence from both the method of incision and the length of incision. Zero variance in some outcomes (e.g., day 30 pain score in the scalpel group)required careful statistical handling. Despite these limitations, the consistency of findings across multiple outcomes strengthens overall conclusions.

## Conclusions

The current study, titled “A study of cutting electrocautery versus scalpel for surgical incisions,” conducted at Gandhi Medical College, Bhopal, drew the following conclusions. After thorough analysis, electrocautery demonstrated clear advantages over scalpel use in terms of intraoperative blood loss, postoperative pain, and wound outcome. Patients in the cautery group consistently experienced reduced blood loss and lower pain levels following surgery. Although the speed of incision placement was similar between both groups, a significant difference in the mean length of incisions was observed, which may have contributed to the differences in the measured outcomes even though the regression model showed a significant difference despite the confounder. On stratification it appears that electrocautery performs better on longer wounds. In spite of a trend suggesting lower complications in the cautery group, no statistically significant difference was observed among the groups. The wound healing was better when using cautery, as a significantly larger number of patients in the scalpel group required secondary procedures for wound management. The study design needs to be strengthened by ensuring better matching of incision length across groups, which would help to reduce the confounding variables. Blood loss was assessed by gain in mop weight, which, although practical, may lack precision compared to direct volumetric measurements.

Despite concerns traditionally associated with cautery-induced thermal injury, our findings suggest that incisions made by electrocautery have significant advantages over scalpel in terms of wound outcome. Electrocautery has a more favorable safety profile than previously assumed. When appropriately used, it offers a tangible benefit and should be considered as a viable and potentially preferable alternative to the scalpel for routine surgical incisions. Future studies with improved study designs and larger sample sizes are required to validate these findings and explore long-term outcomes.
